# Clinical determinants of the PR interval duration in Swiss middle‐aged adults: The CoLaus/PsyCoLaus study

**DOI:** 10.1002/clc.23356

**Published:** 2020-04-24

**Authors:** Marylène Bay, Peter Vollenweider, Pedro Marques‐Vidal, Federica Bocchi, Etienne Pruvot, Jürg Schläpfer

**Affiliations:** ^1^ Department of Medicine, Internal Medicine Lausanne University Hospital (CHUV) Lausanne Switzerland; ^2^ Department of Heart and Vessels, Service of Cardiology Lausanne University Hospital (CHUV) Lausanne Switzerland

**Keywords:** adults, cross‐sectional, determinants, electrocardiogram, PR interval, Switzerland

## Abstract

**Background:**

Prolonged PR interval (PRi) is associated with adverse outcomes. However, PRi determinants are poorly known. We aimed to identify the clinical determinants of the PRi duration in the general population.

**Hypothesis:**

Some clinical data are associated with prolonged PRi.

**Methods:**

Cross‐sectional study conducted between 2014 and 2017. Electrocardiogram‐derived PRi duration was categorized into normal or prolonged (>200 ms). Determinants were identified using stepwise logistic regression, and results were expressed as multivariable‐adjusted odds ratio (OR) (95% confidence interval). A further analysis was performed adjusting for antiarrhythmic drugs, P‐wave contribution to PRi duration, electrolytes (kalemia, calcemia, and magnesemia), and history of cardiovascular disease.

**Results:**

Overall, 3655 participants with measurable PRi duration were included (55.6% females; mean age 62 ± 10 years), and 330 (9.0%) had prolonged PRi. Stepwise logistic regression identified male sex (OR 1.41 [1.02‐1.97]); aging (65‐74 years: OR 2.29 [1.61‐3.24], and ≥ 75 years: OR 4.21 [2.81‐6.31]); increased height (per 5 cm, OR 1.15 [1.06‐1.25]); hypertension (OR 1.37 [1.06‐1.77]); and hs troponin T (OR 1.67 [1.15‐2.43]) as significantly and positively associated, and high resting heart rate (≥70 beats/min, OR 0.43 [0.29‐0.62]) as negatively associated with prolonged PRi. After further adjustment, male sex, aging and increased height remained positively, and high resting heart rate negatively associated with prolonged PRi. Hypertension and hs troponin T were no longer associated.

**Conclusion:**

In a sample of the Swiss middle‐aged population, male sex, aging and increased height significantly increased the likelihood of a prolonged PRi duration, whereas a high resting heart rate decreased it.

## 1 INTRODUCTION

The PR interval (PRi) on the electrocardiogram (ECG) measures the conduction time from the beginning of the P‐wave to the beginning of the QRS complex. It reflects the conduction through the atria, atrioventricular (AV) node, bundle and its branches, and Purkinje fibers.^1^ The normal values range between 120 and 200 millisecond and prolonged PRi or first‐degree atrioventricular block are established when the PRi is >200 millisecond. Prolonged PRi is a frequent ECG finding^2^ that has long been considered as harmless.^3^, ^4^ Yet, one of the studies defining prolonged PRi as benign was based on young and healthy males^4^ and it has been hypothesized that elevated vagal tone and decreased sympathetic tone lead to prolonged PRi, as found in well‐trained athletes.^5^ In 2009, Cheng et al. conducted a study in ambulatory individuals to assess the clinical significance of prolonged PRi. They showed that prolonged PRi was associated with increased risk of atrial fibrillation (AF), all‐cause mortality, and pacemaker implantation.^6^ Consequently, other studies investigated the association between prolonged PRi and several outcomes, including heart failure, cardiovascular mortality or stroke.^7^, ^8^, ^9^, ^10^ Contradictory findings were found, some studies reporting a deleterious effect of prolonged PRi on all‐cause mortality,^8^, ^10^ while others did not.^7^, ^9^

The clinical determinants of prolonged PRi are mostly unknown. Positive associations between the PRi and greater age,^7^, ^10^ BMI,^7^, ^11^ or genetic markers^12^ have been reported. As a prolonged PRi is a frequent finding associated with adverse outcomes, a better identification of the determinants of PRi in an unselected population is recommended. Hence, we aimed to identify the clinical determinants of the PRi duration in the general population.

## 2 METHODS

### 2.1 Study Cohort

The design of the CoLaus study with the detailed baseline and follow‐up methodologies has been reported previously.^13^, ^14^ Briefly, CoLaus is a population‐based prospective study exploring the biological and clinical determinants of cardiovascular diseases. A non‐stratified, representative sample of the population of Lausanne (Switzerland) was recruited between 2003 and 2006, including 6733 participants according to two inclusion criteria: (a) age 35‐75 years, (b) written informed consent. The first follow‐up occurred between 2009 and 2012 and the second between 2014 and 2017. In this cross‐sectional study, all data were collected during the second follow‐up by trained field interviewers and were obtained by a questionnaire, an interview, and a physical examination including blood tests and a 12‐lead digital ECG recording.

### 2.2 Electrocardiography

ECGs were digitally recorded in a resting supine position using a single device (Cardiovit MS‐2015, Schiller AG, Baar, Switzerland). In accordance with the local standards, paper speed was 25 mm/second and calibration 10 mV/mm. Digital ECGs were stored in an anonymised database of SEMA Data Management System (V3.5, Schiller AG, Baar, Switzerland).

ECG measurements were determined by Schiller AG algorithms. As automated measurements of ECG intervals significantly vary between manufacturers^15^ and the diagnostic accuracy of common ECG algorithms is lower than that of cardiologists,^16^ 100 randomly selected ECGs were manually analyzed by M.B. The PRi was defined as the time interval between the earliest detection of atrial depolarization and the earliest detection of ventricular depolarization in any lead. Measurements were performed at a paper speed of 100 mm/second. In case of a > 10 ms disagreement between the automated and the manual values or when diagnoses relative to the PRi (eg, sinus rhythm, AF) were discordant, a senior cardiologist (J.S.) reanalyzed the ECG and measured the PRi. This procedure showed a good agreement between the PRi durations assessed digitally and manually, except for the three following conditions: (a) extreme digital PRi durations (>2 or < 2 SD, respectively >220 ms or < 116 ms); (b) non sinus rhythm or AV conduction abnormality; and (c) missing of PRi duration in presence of sinus rhythm. Hence, in this study, manual analyzes were performed for these three conditions (corresponding to 475 ECGs, ie, 13% of the ECGs). The analyzes were conducted by two investigators (M.B., F.B.) and further confirmed by two senior cardiologists (J.S., E.P.). For the remaining ECGs, digitally determined PRi durations were used. PRis were then categorized into prolonged (>200 ms) or normal (≤200 ms) for analysis.

### 2.3 Clinical data

Age was categorized in four 10‐year groups (45‐54 years, 55‐64 years, 65‐74 years and > 75 years).

Body weight and height were measured with participants barefoot and in light indoor clothes. Body weight was measured to the nearest 100 g using a Seca scale (Hamburg, Germany) and height was measured to the nearest millimeter using a Seca height gage. Obesity was defined as a body mass index (BMI) ≥30 kg/m^2^ and overweight as BMI ≥25 kg/m^2^ and < 30 kg/m^2^. Waist circumference was measured midway between the lowest rib and the iliac crest using a non‐stretchable tape and the average of two measurements was taken. Abdominal obesity was defined as a waist circumference ≥ 102 cm (men) and ≥ 88 cm (women).^17^

Alcohol consumption and smoking status were assessed by self‐filled questionnaire. Excessive alcohol consumption was defined as >40 g/day for men and > 20 g/day for women.^18^ Participants were considered as current or former smokers when reporting smoking (any type of tobacco combustion), and nonsmoking otherwise.

Cardiovascular risk assessment was evaluated with two risk equations, the European Society of Cardiology SCORE^19^ recalibrated for Switzerland^20^ and the IAS‐arbeitsgruppe lipide und atherosklerose (AGLA) score.^21^ The SCORE risk estimates the 10‐year risk of death from vascular causes and the AGLA risk estimates the 10‐year risk of nonfatal myocardial infarction.

Resting heart rate was obtained on the ECG and defined as high when ≥70 beats per minute.^22^ Blood pressure (BP) was measured after at least a 10‐minute rest in a seated position using an Omron HEM‐907 automated oscillometric sphygmomanometer with an appropriately sized cuff. Three measurements separated by 10‐minute intervals were performed and the average of the last two measurements was used. Hypertension was defined by a systolic BP ≥140 mmHg and/or a diastolic BP ≥90 mmHg and/or presence of antihypertensive treatment.

History of cardiovascular disease (CVD) included myocardial infarction, angina pectoris, percutaneous revascularization or bypass grafting, stroke or transient ischemic attack. History of CVD was obtained either based on patient's report (for some of the events occurring before the baseline CoLaus survey) or based on clinical data (obtained during follow‐up) validated by an independent adjudication committee including cardiologists and a neurologist.^14^

Participants listed their medications in the self‐filled questionnaire. Antiarrhythmic drugs including digoxin, calcium channel blockers (CCBs), amiodarone, and beta‐blockers were selected for adjustment because of their impact on the PRi.

### 2.4 Biological data

Fasting venous blood samples were processed in the Lausanne University Hospital laboratory. Biological parameters included glucose; HbA1c; total, HDL and LDL‐cholesterol; triglycerides; creatinine; NT‐proBNP; high‐sensitivity cardiac troponin T (hs cTnT), and electrolytes (magnesium, potassium, calcium) for their effect on cardiac conduction.

Diabetes mellitus was defined as fasting plasma glucose ≥7.0 mmoL/L and/or HbA1c ≥48 mmol/mol (≥6.5%) and/or anti‐diabetic treatment. Renal failure was defined by eGFR <60 mL/min/1.73 m^2^ (1 mL/s/m^2^) using the CKD‐EPI formula. Dyslipidemia was defined either by using the LDL‐cholesterol thresholds adapted from the Systematic Coronary Risk Evaluation (SCORE) risk charts (Table S1), and/or by presence of a lipid lowering treatment. Elevated NT‐proBNP was considered when ≥125 ng/L and elevated hs cTnT when ≥14 ng/L (≥0.014 μg/L).

### 2.5 Exclusion criteria

**Figure 1:**
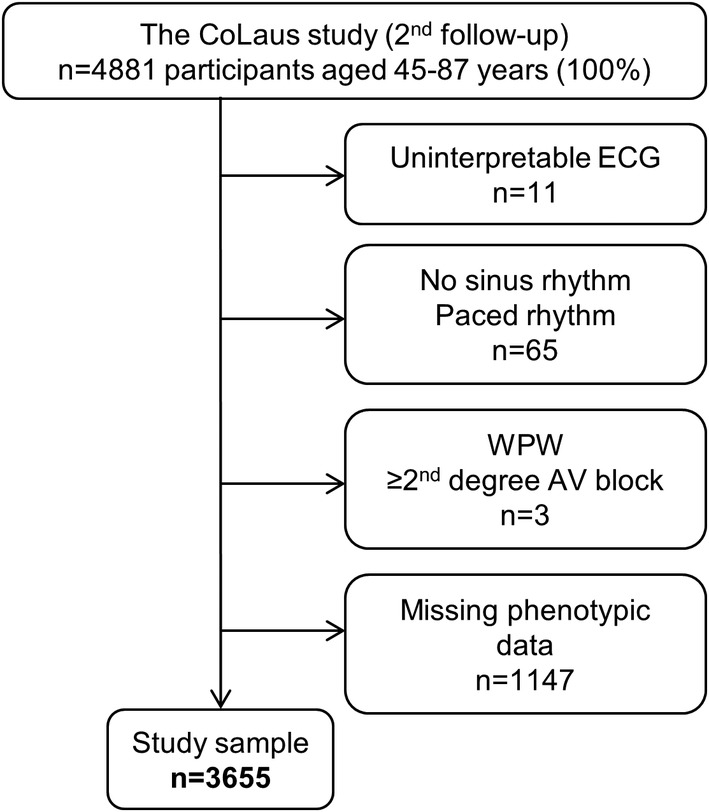
Flow diagram: participants selection procedure. AV, atrioventricular; WPW, Wolff‐Parkinson‐White

Exclusion criteria for the current analyzes were as follows: (a) uninterpretable ECG (ie, unstable baseline, missing or inverted electrodes); (b) no sinus rhythm or paced rhythm; (c) Wolff‐Parkinson‐White syndrome or ≥ second degree AV block; and (d) missing phenotypic data (Figure 1).

### 2.6 Statistical analyzes

Statistical analyzes were conducted using STATA version 15.1 for Windows (Stata Corp, College Station, Texas). Concordance between automatic and manual PRi measurements was assessed by Spearman correlation and Lin's concordance coefficients.

Bivariate analysis of the factors associated with prolonged PRi was performed using chi‐square for qualitative variables and Student's *t*‐test for continuous variables. Results were expressed as number of participants (percentage) or as average ± SD. Multivariable analysis using the PRi duration as dependent variable was performed by stepwise forward logistic regression and findings were further confirmed by stepwise backward logistic regression. Results were expressed as odds ratio (OR) and 95% confidence interval (CI).

Model 1 tested the following covariates: sex; age (45‐54, 55‐64, 65‐74, 75+ years); height (continuous); BMI (normal, overweight, obese); waist (normal, elevated); alcohol intake (none, moderate, excessive); smoking status (never, former, current); 10‐year risk of coronary heart disease (CHD) (SCORE and AGLA: low, middle, high, very high); diabetes mellitus (yes/no); hypertension (yes/no); dyslipidemia (yes/no); renal insufficiency (yes/no); resting heart rate (<70, ≥70 bpm); hs cTnT (<14, ≥14 ng/L) and NT‐proBNP (<125, ≥125 ng/L). Model 2 tested the same set of variables as model 1, but adjusting for antiarrhythmic drugs; electrolytes (magnesium, potassium, calcium); P‐wave contribution to the length of the PRi (P duration/PR duration×100 as suggested by Soliman and et al.^23^) and history of CVD. Model 3 included the same covariates as model 2, but participants under beta‐blockers and non‐cardioselective CCBs (ATC C08C, C08E, and C08G) were excluded.

Sensitivity analyzes were conducted using inverse probability weighting. Briefly, a logistic model was built including variables significantly different between included and excluded participants, and the probability of inclusion was computed.^24^ The inverse of the probability that the observation is included was then used as weight in the different models described above. A second sensitivity analysis was conducted using age and heart rate as continuous variables. Statistical significance was defined by a two‐sided *P*‐value <.05.

### 2.7 Ethical statement and consent

The local Institutional Ethics Committee approved the baseline CoLaus study (reference 16/03, decisions of 13 January and 10 February 2003); the approval was renewed for the first (reference 33/09, decision of 23 February 2009) and the second (reference 26/14, decision of 11 March 2014) follow‐up. The study was performed in agreement with the Helsinki declaration and the applicable Swiss legislation. All participants gave their signed informed consent before entering the study.

## 3 RESULTS

### 3.1 Concordance between computerized and manual ECG analyzes

Spearman's rho was 0.95 and concordance correlation coefficient 0.95 (both *P* < .001). The digital algorithm related to the PRi was incorrect for two ECGs: (a) the digital diagnosis was sinus rhythm with an extremely long PRi, while the correct manual diagnosis was AF; (b) the digital diagnosis was an irregular rhythm with no P‐wave detected, while the correct manual diagnosis was sinus rhythm. Furthermore, P‐wave and PR values were missing in a correctly diagnosed case of sinus bradycardia.

### 3.2 Study population

Of the initial 4881 participants, 1226 (25.1%) were excluded. The reasons for exclusion are shown in Figure 1 and the characteristics of excluded and included participants are summarized in Table S2. Excluded participants were older, shorter, with higher BMI, had more abdominal obesity, excessive alcohol intake, diabetes, renal insufficiency, elevated CHD risk scores, dyslipidemia, hypertension, high resting heart rate and elevated hs cTnT and NT‐proBNP than included ones.

### 3.3 Factors associated with prolonged PRi

Of the 3655 participants with interpretable ECG and measurable PRi duration, 330 (9.0%, 95% CI 8.1 to 10.0%) presented with a prolonged PRi. The clinical characteristics of the participants, overall and according to categories of PRi duration, are presented in Table 1. Participants with prolonged PRi were more frequently male, old, tall and obese. They also had a higher prevalence of renal failure, dyslipidemia, elevated CHD risk scores, hypertension and elevated hs cTnT and NT‐proBNP levels. Inversely, they were less prone to smoke and to have high resting heart rate.

**Table 1:** Clinical characteristics of the participants, overall and according to PR interval duration, CoLaus/PsyCoLaus study, Lausanne, Switzerland, 2014‐2017

	Overall	PR ≤ 200 ms	PR > 200 ms	*P*‐value
No.	3655	3325	330	
Female sex (%)	2032 (55.6)	1900 (57.1)	132 (40.0)	<.001
Age (y)	61.8 ± 9.9	61.3 ± 9.7	66.6 ± 10.6	<.001
Age categories (y) (%)				<.001
45–54	1096 (29.9)	1035 (31.1)	61 (18.5)	
55–64	1201 (32.9)	1128 (33.9)	73 (21.1)	
65–74	944 (25.8)	834 (25.1)	110 (33.3)	
75+	414 (11.3)	328 (9.9)	86 (26.1)	
Height (cm)	167.8 ± 9.5	167.5 ± 9.4	170.1 ± 10.3	<.001
Body mass index (kg/m^2^)	26.2 ± 4.6	26.2 ± 4.7	26.8 ± 4.1	.02
Body mass index categories				.03
Normal	1572 (43.0)	1452 (43.7)	120 (36.4)	
Overweight	1445 (39.5)	1302 (39.2)	143 (43.3)	
Obese	638 (17.5)	571 (17.2)	67 (20.3)	
Abdominal obesity (%)	1310 (35.8)	1176 (35.4)	134 (40.6)	.06
Alcohol intake (%)				.34
None	951 (26.0)	858 (25.8)	93 (28.2)	
Moderate	2492 (68.2)	2269 (68.2)	223 (67.6)	
Excessive	212 (5.8)	198 (5.9)	14 (4.2)	
Smoking (%)				.009
Never	1546 (42.3)	1396 (41.9)	150 (45.5)	
Former	1426 (39.0)	1287 (38.7)	139 (42.1)	
Current	683 (18.7)	642 (19.3)	41 (12.4)	
Diabetes mellitus (%)	334 (9.1)	295 (8.9)	39 (11.8)	.08
Renal failure (%)	291 (7.9)	247 (7.4)	44 (13.3)	<.001
10 year risk of CHD (SCORE)				<.001
Low <1%	1124 (30.8)	1066 (32.1)	58 (17.6)	
Medium (≥1 to <5%)	1397 (38.2)	1281 (38.5)	116 (35.2)	
High (≥5 to <10%)	698 (19.1)	599 (18.0)	99 (30.0)	
Very high (≥10%)	436 (11.9)	379 (11.4)	57 (17.3)	
Dyslipidemia (SCORE) (%)	1588 (43.5)	1404 (42.2)	184 (55.8)	<.001
10 year risk of CHD (AGLA)				<.001
Low (<10%)	2582 (70.6)	2385 (71.7)	197 (59.7)	
Middle (10‐19%)	142 (3.9)	124 (3.7)	18 (5.5)	
High (≥20%)	83 (2.3)	69 (2.1)	14 (4.2)	
Very high	848 (23.2)	747 (22.5)	101 (30.6)	
Hypertension (%)	1588 (43.5)	1393 (41.9)	195 (59.1)	<.001
Elevated (≥70 bpm) resting heart rate (%)	650 (17.8)	617 (18.6)	33 (10.0)	<.001
Elevated (≥14 ng/L) hs cTnT (%)	236 (6.5)	181 (5.4)	55 (16.7)	<.001
Elevated (≥125 ng/L) NT‐proBNP (%)	744 (20.4)	639 (19.2)	105 (31.8)	<.001

*Note*: Results are expressed as mean ± SD or as number of participants (percentage). Between‐group comparisons using chi‐square or student *t* test.

Abbreviations: AGLA, Arbeitsgruppe Lipide und Atherosklerose; bpm, beats per minute; CHD, coronary heart disease; hs cTnT, high‐sensitivity cardiac troponin T.

Table 2 displays the results of the multivariable stepwise logistic regression assessing the associations between prolonged PRi and clinical characteristics. In model 1, male sex, older age, increased height, hypertension and elevated hs cTnT were significantly and positively associated with prolonged PRi, while high resting heart rate was negatively associated.

**Table 2:** Multivariable associations between prolonged (>200 msec) PR interval and clinical characteristics of participants, CoLaus/PsyCoLaus study, Lausanne, Switzerland, 2014‐2017

	Model 1 (n = 3655)		Model 2 (n = 3397)		Model 3 (n = 2991)	
	OR (95% CI)	*P*‐value	OR (95% CI)	*P*‐value	OR (95% CI)	*P*‐value
Sex						
Female	1 (ref.)		1 (ref.)		1 (ref.)	
Male	1.41 (1.02‐1.97)	.040	1.78 (1.20‐2.66)	.005	2.14 (1.36‐3.35)	.001
Age (y)						
45‐54	1 (ref.)		1 (ref.)		1 (ref.)	
55‐64	1.11 (0.77‐1.58)	.582	1.21 (0.80‐1.82)	.368	1.17 (0.76‐1.82)	.47
65‐74	2.29 (1.61‐3.24)	<.001	2.42 (1.60‐3.68)	<.001	2.67 (1.70‐4.19)	<.001
75+	4.21 (2.81‐6.31)	<.001	5.12 (3.19‐8.21)	<.001	5.39 (3.16‐9.21)	<.001
*P*‐value for trend	<.001		<.001		<.001	
Height (per 5 cm)	1.15 (1.06‐1.25)	.001	1.23 (1.12‐1.37)	<.001	1.26 (1.12‐1.42)	<.001
Hypertension						
No	1 (ref.)		Not retained		Not retained	
Yes	1.37 (1.06–1.77)	.015				
Resting heart rate						
Normal (<70 bpm)	1 (ref.)		1 (ref.)		1 (ref.)	
Elevated (≥70 bpm)	0.43 (0.29‐0.62)	<.001	0.54 (0.34‐0.85)	.007	0.44 (0.25‐0.77)	.004
Hs cTnT categories						
Normal (<14 ng/L)	1 (ref.)		Not retained		Not retained	
Elevated (≥14 ng/L)	1.67 (1.15‐2.43)	.007				

*Note*: Results are expressed as multivariable‐adjusted odds ratio (95% confidence interval). Analysis by stepwise forward logistic regression; results were further confirmed by stepwise backward logistic regression. Model 1 included all variables from Table 1 except for age and BMI as continuous variables. Model 2 tested the same set of variables as model 1, but adjusting for antiarrhythmic drugs; electrolytes (magnesium, potassium, calcium); P‐wave contribution to the length of the PR interval (P duration/PR duration×100) and history of CVD. Model 3 included the same covariates as in model 2, but participants under beta‐blockers and non‐cardioselective CCBs were excluded.

Abbreviations: bpm, beats per minute; CI, confidence interval; Hs cTnT, high‐sensitivity cardiac troponin T, OR, odds ratio .

After further adjustment according to model 2, male sex, older age, and increased height remained positively, and high resting heart rate negatively associated with prolonged PRi. Conversely, hypertension and hs cTnT were no longer associated. Results were similar after exclusion of participants under beta‐blockers and non‐cardioselective CCBs (model 3).

### 3.4 Sensitivity analysis

The results of the sensitivity analysis using inverse probability weighting are summarized in Table S3. The factors retained were identical to those of the initial analyzes. Similar findings were obtained using age and heart rate as continuous variables (Table S4).

## 4 DISCUSSION

In this study, male sex, older age and increased height were significantly and positively associated with prolonged (>200 ms) PRi, while high resting heart rate was negatively associated. These associations were independent of the P‐wave contribution to the length of PRi.

### 4.1 Agreement between computerized and manual ECG analyzes

The concordance between manual and digital measures of PRi duration and PRi‐related diagnoses was good. It has been demonstrated that errors in digital ECG diagnoses are frequently related to arrhythmia and conduction disorders.^16^ In our study, there were two incorrect ECG diagnoses by the digital algorithm: one sinus rhythm case misdiagnosed as AF, and one AF case misdiagnosed as an extremely long PRi. In summary, our ECG digital data were reliable for epidemiological studies, but a validation of the algorithm on ECGs sample, and a manual reading is recommended for the following conditions: (a) extreme digital PRi durations (> or < 2 SD); (b) non sinus rhythm or AV conduction abnormality; and (c) absence of PRi duration when sinus rhythm is reported.

### 4.2 Prevalence of prolonged PRi

In our sample, approximately one out of 11 (9.0%, 95% CI 8.1‐10.0) participants had a prolonged PRi. This is in mid‐range of other studies reporting prevalence rates ranging from 1.6% to 18%.^6^, ^7^, ^9^, ^11^, ^23^ Several explanations may help to explain these differences. First, by the different characteristics of the studied populations; for example, Holmqvist et al.^11^ reported an 18% prevalence rate of prolonged PRi but participants with established coronary artery disease were included, a condition known to increase the risk of prolonged PRi. Second, by different age; Cheng et al.^6^ reported a low (1.6%) prevalence rate in a sample with a mean age of 47 years compared to >60 years in the present study. Conversely, a study reporting prevalence rate of prolonged PRi in a population similar to CoLaus showed a comparable result (8.7%).^23^

### 4.3 Factors associated with prolonged PRi

Older age was positively associated with prolonged PRi, participants aged >75 years having a more than fourfold increase in the likelihood of prolonged PRi compared to the youngest age category. Similar findings were obtained when age was used as a continuous variable. This is a consistent finding in the literature.^7^, ^10^, ^11^ A major explanation is that fibrosis increases in the aging heart due to inflammation, haemodynamic factors, cellular senescence and death, and reactive oxygen species^25^ and, subsequently, increased fibrosis slows cardiac conduction leading to prolonged PRi.^26^

Male sex was positively associated with prolonged PRi, a finding also reported elsewhere.^7^, ^11^ The reasons for this association are not completely understood. It has been proposed that men have a larger heart size, implicating a longer His‐Purkinje system and hence a prolonged conduction time.^27^ Sex hormones might also be implicated: an animal study has demonstrated that estrogen attenuates the pro‐myofibroblast proliferation effect of angiotensin II,^28^ thus reducing cardiac fibrosis. Nevertheless, the reasons why male sex increases the likelihood of prolonged PRi are still speculative and deserve further investigations.

A 5 cm increase in height increased the likelihood of a prolonged PRi by 26%. To our knowledge, height has been seldom associated with ECG characteristics. Nonetheless, both the PRi and height have been linked with AF,^6^, ^29^ and Kofler et al.^30^ recently observed significant associations of measured and genetically determined height with PRi suggesting that “adult height is a marker of altered cardiac conduction and that these relationships might be causal.”^30^ Our results support this hypothesis. However, the commonly advanced explanation that tall persons also have a larger heart, which causes PRi prolongation is now debated.^30^

High resting heart rate was the only factor associated with a reduced likelihood of a prolonged PRi, a finding also reported elsewhere.^7^ A plausible explanation is that sympathetic activity increases heart rate by shortening the cardiac conduction cycle, partly by accelerating the AV node conduction.^31^

Hypertension and elevated hs cTnT were positively but inconsistently associated with prolonged PRi. A possible explanation for hypertension not being retained in model 2 is linked to the adjustment for medication. As hypertension was defined partly by the presence of antihypertensive drugs (beta‐blockers and CCBs included), adjusting for antihypertensive drugs reduced the strength of the association. Still, hypertension was not retained even after excluding participants on beta‐blockers and non‐cardioselective CCBs. This echoes the contradictory findings of the literature, where significant^11^ or non‐significant^7^, ^9^ associations between hypertension and PRi duration have been reported. Similarly, hs cTnT was inconsistently associated with PRi duration, possibly because of the adjustment for CVD history. Yet, and despite the inconsistent statistical findings, we believe that hypertension and hs cTnT might be associated with prolonged PRi as both increase the risk of cardiac fibrosis.^25^, ^32^

### 4.4 Limitations

This study has several limitations. First, it was limited to an age range of 45 to 86, and might not be applicable in younger or older participants. Second, the sample was mostly restricted to Caucasians and might not be generalizable to other ethnicities. Third, a sizable fraction (one‐quarter) of the sample was excluded, and excluded participants differed from the included ones regarding the levels of several determinants of prolonged PRi; this might have biased the associations between potential determinants and prolonged PRi. Still, the results obtained were almost identical when inverse probability weighting was applied. Finally, most PRi durations were digitally measured and errors may have occurred. However, we endeavored to control the reliability of the digital analyzes and optimize the manual reading.

### 4.5 Conclusion

In a sample of the Swiss middle‐aged population, male sex, older age, and increased height significantly increased the likelihood of a prolonged PRi duration, whereas high resting heart rate decreased it. The effect of hypertension and elevated hs cTnT on the PRi duration needs further investigations.

## CONFLICT OF INTEREST

The authors declare no potential conflict of interest.

## AUTHOR CONTRIBUTIONS

M.B., P.M.V., P.V. and J.S. designed the present study; all authors were involved in data collection; M.B. drafted the manuscript; P.V., P.M.V., F.B., E.P and J.S. critically revised the manuscript. All authors gave final approval.

The funding source was not involved in the study design, data collection, analysis and interpretation, writing of the report, or decision to submit the article for publication.

## Supporting information


**Table S1** LDL cholesterol levels considered for the definition of dyslipidemia, according to total cardiovascular risk (SCORE).
Table s2: Characteristics of excluded and included participants, CoLaus/PsyCoLaus, Lausanne, Switzerland, 2014‐2017.
Table s3: Sensitivity analysis conducted using inverse probability weighting, CoLaus/PsyCoLaus study, Lausanne, Switzerland, 2014‐2017.
Table S4: Sensitivity analysis conducted using inverse probability weighting with age and heart as continuous variables, CoLaus/PsyCoLaus study, Lausanne, Switzerland, 2014‐2017.
