# Supplementary material for: Clinical determinants of the PR interval duration in Swiss middle‐aged adults: The CoLaus/PsyCoLaus study
Source: Clin Cardiol. 2020 Apr 24;43(6):614–21. doi: 10.1002/clc.23356 (PMC7299001; doi:10.1002/clc.23356)
Supplement: Supplementary file 1 — Table S1 LDL cholesterol levels considered for the definition of dyslipidemia, according to total cardiovascular risk (SCORE). Table s2: Characteristics of excluded and included participants, CoLaus/PsyCoLaus, Lausanne, Switzerland, 2014‐2017. Table s3: Sensitivity analysis conducted using inverse probability weighting, CoLaus/PsyCoLaus study, Lausanne, Switzerland, 2014‐2017. Table S4: Sensitivity analysis conducted using inverse probability weighting with age and heart as continuous variables, CoLaus/PsyCoLaus study, Lausanne, Switzerland, 2014‐2017. [file CLC-43-614-s001.docx]

**Clinical determinants of the PR interval duration in Swiss middle-aged adults: the CoLaus/PsyCoLaus study**

M. Bay; P. Vollenweider; P. Marques-Vidal; F. Bocchi; E. Pruvot; J. Schläpfer

**Online-Only : eTABLES**

Table of contents

[eTable 1: LDL cholesterol levels considered for the definition of dyslipidemia, according to total cardiovascular risk (SCORE). 2](#_Toc32347692)

[eTable 2: Characteristics of excluded and included participants, CoLaus/PsyCoLaus, Lausanne, Switzerland, 2014-2017. 3](#_Toc32347693)

[eTable 3: Sensitivity analysis conducted using inverse probability weighting, CoLaus/PsyCoLaus study, Lausanne, Switzerland, 2014-2017. 5](#_Toc32347694)

[eTable 4: Sensitivity analysis conducted using inverse probability weighting with age and heart as continuous variables, CoLaus/PsyCoLaus study, Lausanne, Switzerland, 2014-2017. 7](#_Toc32347695)

# **eTable 1**: LDL cholesterol levels considered for the definition of dyslipidemia, according to total cardiovascular risk (SCORE).

| **Total CV risk (%)** | **Intervention levels (mmol/L)** |
| --- | --- |
| <1 | ≥4.9 |
| ≥1 to <5 | ≥4.0 |
| ≥5 to <10 or high-risk | ≥2.6 |
| ≥10 or high-risk | ≥1.8 |

Adapted from Catapano AL et al. 2016 ESC/EAS Guidelines for the Management of Dyslipidaemias. Eur Heart J. 2016;37(39):2999-3058.

# **eTable 2**: Characteristics of excluded and included participants, CoLaus/PsyCoLaus, Lausanne, Switzerland, 2014-2017.

|  | **Included**  **(n=3655)** | **Excluded**  **(n=1226)** | **p-value** |
| --- | --- | --- | --- |
| Prolonged (>200 ms) PR interval (%) | 330 (9.0) | 66 (10.6) | 0.204 |
| Women (%) | 2032 (55.6) | 657 (53.6) | 0.222 |
| Age (years) | 61.8 ± 9.9 | 66.5 ± 11.2 | <0.001 |
| Age categories (years) (%) |  |  | <0.001 |
| 45-54 | 1096 (29.9) | 250 (20.4) |  |
| 55-64 | 1201 (32.9) | 301 (24.6) |  |
| 65-74 | 944 (25.8) | 355 (28.9) |  |
| 75+ | 414 (11.3) | 320 (26.1) |  |
| Height (cm) | 167.8 ± 9.5 | 166.4 ± 9.4 | <0.001 |
| Body mass index (kg/m^2^) | 26.2 ± 4.6 | 27.3 ± 4.9 | 0.019 |
| Body mass index categories (%) |  |  | <0.001 |
| Normal | 1572 (43.0) | 285 (34.5) |  |
| Overweight | 1445 (39.5) | 330 (39.9) |  |
| Obese | 638 (17.5) | 211 (25.5) |  |
| Abdominal obesity (%) | 1310 (35.8) | 383 (46.3) | <0.001 |
| Alcohol intake (%) |  |  | <0.001 |
| None | 951 (26.0) | 224 (38.3) |  |
| Moderate | 2492 (68.2) | 323 (55.2) |  |
| Excessive | 212 (5.8) | 38 (6.5) |  |
| Smoking (%) |  |  | 0.429 |
| Never | 1546 (42.3) | 340 (40.5) |  |
| Former | 1426 (39.0) | 328 (39.1) |  |
| Current | 683 (18.7) | 172 (20.5) |  |
| Diabetes mellitus (%) | 334 (9.1) | 198 (22.3) | <0.001 |
| Renal failure (%) | 291 (7.9) | 138 (16.1) | <0.001 |
| 10-year risk of CHD (SCORE) (%) |  |  | <0.001 |
| Low <1% | 1124 (30.8) | 90 (13.0) |  |
| Medium (≥1 to <5%) | 1397 (38.2) | 150 (21.7) |  |
| High (≥5 to <10%) | 698 (19.1) | 229 (33.1) |  |
| Very high (≥10%) | 436 (11.9) | 223 (32.2)) |  |
| Dyslipidemia (SCORE) (%) | 1588 (43.5) | 535 (58.0) | <0.001 |

|  | **Included**  **(n=3655)** | **Excluded**  **(n=1226)** | **p-value** |
| --- | --- | --- | --- |
| 10-year risk of CHD (AGLA) (%) |  |  | <0.001 |
| Low (<10%) | 2582 (70.6) | 253 (36.1) |  |
| Middle (10-19%) | 142 (3.9) | 20 (2.9) |  |
| High (≥20%) | 83 (2.3) | 20 (2.9) |  |
| Very high | 848 (23.2) | 408 (58.2) |  |
| Hypertension (%) | 1588 (43.5) | 675 (65.5) | <0.001 |
| Elevated (≥70 bpm) resting heart rate (%) | 650 (17.8) | 194 (28.4) | <0.001 |
| Elevated (≥14 ng/l) hs cTnT (%) | 236 (6.5) | 146 (17.5) | <0.001 |
| Elevated (≥125 ng/l) NT-proBNP (%) | 744 (20.4) | 323 (37.9) | <0.001 |

SI conversion factors: To convert cTnT in µg/l, divide by 1000. AGLA, Arbeitsgruppe Lipide und Atherosklerose; bpm, beats per minute; CHD, coronary heart disease; hs cTnT, high-sensitivity cardiac troponin T.

Results are expressed as mean ± SD or as number of participants (percentage). Between-group comparisons using chi-square or student t-test. For excluded participants, numbers might not add to the total number due to missing values.

# **eTable 3**: Sensitivity analysis conducted using inverse probability weighting, CoLaus/PsyCoLaus study, Lausanne, Switzerland, 2014-2017.

|  | **Model 1 (n=3655)** | | **Model 2 (n=3397)** | | **Model 3 (n=2991)** | |
| --- | --- | --- | --- | --- | --- | --- |
|  | **OR (95% CI)** | **p-value** | **OR (95% CI)** | **p-value** | **OR (95% CI)** | **p-value** |
| Sex |  |  |  |  |  |  |
| Female | 1 (ref.) |  | 1 (ref.) |  | 1 (ref.) |  |
| Male | 1.42 (1.04 - 1.94) | 0.028 | 1.74 (1.17 - 2.58) | 0.006 | 2.11 (1.34 - 3.32) | 0.001 |
| Age (years) |  |  |  |  |  |  |
| 45-54 | 1 (ref.) |  | 1 (ref.) |  | 1 (ref.) |  |
| 55-64 | 1.13 (0.80 - 1.60) | 0.493 | 1.24 (0.83 - 1.86) | 0.285 | 1.18 (0.76 - 1.83) | 0.466 |
| 65-74 | 2.27 (1.62 - 3.18) | <0.001 | 2.38 (1.57 - 3.60) | <0.001 | 2.69 (1.73 - 4.21) | <0.001 |
| 75+ | 4.13 (2.77 - 6.16) | <0.001 | 4.94 (3.09 - 7.91) | <0.001 | 5.43 (3.14 - 9.38) | <0.001 |
| p-value for trend | <0.001 |  | <0.001 |  | <0.001 |  |
| Height (per 5 cm) | 1.15 (1.06 - 1.25) | 0.001 | 1.24 (1.12 - 1.38) | <0.001 | 1.27 (1.13 - 1.42) | <0.001 |
| Hypertension |  |  |  |  |  |  |
| No | 1 (ref.) |  | Not retained |  | Not retained |  |
| Yes | 1.39 (1.08 - 1.80) | 0.011 |  |  |  |  |

|  | **Model 1 (n=3655)** | | **Model 2 (n=3397)** | | **Model 3 (n=2991)** | |
| --- | --- | --- | --- | --- | --- | --- |
|  | **OR (95% CI)** | **p-value** | **OR (95% CI)** | **p-value** | **OR (95% CI)** | **p-value** |
| Resting heart rate |  |  |  |  |  |  |
| Normal (<70 bpm) | 1 (ref.) |  | 1 (ref.) |  | 1 (ref.) |  |
| Elevated (≥70 bpm) | 0.45 (0.31 - 0.66) | <0.001 | 0.57 (0.35 - 0.94) | 0.027 | 0.43 (0.24 - 0.76) | 0.004 |
| Hs cTnT categories |  |  |  |  |  |  |
| Normal (<14 ng/l) | 1 (ref.) |  | Not retained |  | Not retained |  |
| Elevated (≥14 ng/l) | 1.72 (1.16 - 2.55) | 0.006 |  |  |  |  |

SI conversion factors: To convert cTnT in µg/l, divide by 1000. OR, Odds ratio; Hs cTnT, high-sensitivity cardiac troponin T.

Results are expressed as multivariable-adjusted odds ratio (95% confidence interval). The logistic model was built including variables significantly different between included and excluded participants, and the probability of inclusion was computed. The inverse of the probability that the observation is included was then used as weight in the different models described above.

# **eTable 4**: Sensitivity analysis conducted using inverse probability weighting with age and heart as continuous variables, CoLaus/PsyCoLaus study, Lausanne, Switzerland, 2014-2017.

|  | **Model 1 (n=3655)** | | **Model 2 (n=3397)** | | **Model 3 (n=2991)** | |
| --- | --- | --- | --- | --- | --- | --- |
|  | **OR (95% CI)** | **p-value** | **OR (95% CI)** | **p-value** | **OR (95% CI)** | **p-value** |
| Sex |  |  |  |  |  |  |
| Female | 1 (ref.) |  | 1 (ref.) |  | 1 (ref.) |  |
| Male | 1.35 (0.99 - 1.84) | 0.057 | 1.7 (1.15 - 2.52) | 0.008 | 1.99 (1.27 - 3.11) | 0.003 |
| Age (years) | 1.05 (1.04 - 1.07) | <0.001 | 1.06 (1.04 - 1.08) | <0.001 | 1.07 (1.05 - 1.09) | <0.001 |
| Height (per 5 cm) | 1.15 (1.06 - 1.26) | 0.001 | 1.25 (1.13 - 1.39) | <0.001 | 1.29 (1.16 - 1.45) | <0.001 |
| Resting heart rate (per 10 bpm) | 0.63 (0.54 - 0.75) | <0.001 | 0.70 (0.56 - 0.88) | 0.002 | 0.64 (0.52 - 0.78) | <0.001 |
| Hypertension |  |  |  |  |  |  |
| No | 1 (ref.) |  | Not retained |  | Not retained |  |
| Yes | 1.42 (1.10 - 1.83) | 0.007 |  |  |  |  |
| Hs cTnT categories |  |  |  |  |  |  |
| Normal (<14 ng/l) | 1 (ref.) |  | Not retained |  | Not retained |  |
| Elevated (≥14 ng/l) | 1.81 (1.22 - 2.67) | 0.003 |  |  |  |  |

OR, Odds ratio; Hs cTnT, high-sensitivity cardiac troponin T.

Results are expressed as multivariable-adjusted odds ratio (95% confidence interval). The logistic model was built including variables significantly different between included and excluded participants, and the probability of inclusion was computed. The inverse of the probability that the observation is included was then used as weight in the different models described above.
